# Evaluation of the tolerability and safety of [^225^Ac]Ac-PSMA-I&T in patients with metastatic prostate cancer: a phase I dose escalation study

**DOI:** 10.1186/s12885-024-11900-y

**Published:** 2024-01-29

**Authors:** Sui wai Ling, Astrid A. M. van der Veldt, Mark Konijnenberg, Marcel Segbers, Eline Hooijman, Frank Bruchertseifer, Alfred Morgenstern, Erik de Blois, Tessa Brabander

**Affiliations:** 1 Department of Radiology & Nuclear Medicine, Erasmus MC, Rotterdam, The Netherlands; 2 Department of Medical Oncology, Erasmus MC, Rotterdam, The Netherlands; 3 Department of Hospital Pharmacy, Erasmus MC, Rotterdam, The Netherlands; 4 European Commission, Joint Research Centre (JRC), Karlsruhe, Germany

**Keywords:** Prostate cancer, Actinium-225, PSMA I&T, Clinical protocol, Dose escalation

## Abstract

**Background:**

Life expectancy of patients with metastatic castration-resistant prostate cancer (mCRPC) is still limited despite several systemic treatments. Within five years after diagnosis of primary prostate cancer, 10–20% of the patients have mCRPC and curation is not an option. Radionuclide therapy (RNT) targeted against prostate-specific membrane antigen (PSMA) emerged as a new treatment option and showed effective results in patients with mCRPC. Survival benefit after [^177^Lu]Lu-PSMA RNT has already been demonstrated in several clinical trials. However, [^225^Ac]Ac-PSMA (^225^Ac-PSMA) appears to be an even more promising radiopharmaceutical for the treatment of mCRPC. The use of alpha emitting radionuclides offers advantages over beta emitting radionuclides due to the high linear energy transfer effective for killing tumor cells and the limited range to reduce the radiation effects on the healthy tissue. However, these results are based on retrospective data and safety data of ^225^Ac-PSMA are still limited. Therefore, a prospective trial is needed to determine the optimal amount of activity that can be administered.

**Methods:**

The ^225^Ac-PSMA-Imaging & Therapy (I&T) trial is an investigator-initiated phase I, single-center, open label, repeated dose-escalation and expansion trial. Patient with PSMA-positive mCRPC after at least one line of chemotherapy and/or one line of nonsteroidal antiandrogen will be treated with ^225^Ac-PSMA-I&T in increasing amount of activity per cycle. Dose-escalation following an accelerated 3 + 3 design which allows to open the next dose-level cohort in the absence of dose limiting toxicity while the previous one is still ongoing. Up to 4 treatment cohorts will be explored including up to 3 dose-escalation cohorts and one expansion cohort where patients will be administered with the recommended dose. A total of up to 30 patients will be enrolled in this trial. All patients will be evaluated for safety. Additionally, dosimetry was performed for the patients in the dose-escalation cohorts after the first ^225^Ac-PSMA-I&T administration.

**Discussion:**

This trial will assess the safety and tolerability of ^225^Ac-PSMA-I&T in patients with mCRPC to recommend the optimal dose for the phase II trial.

**Trial registration:**

ClinicalTrials.gov, (NCT05902247). Retrospectively registered 13 June 2023.

## Background

Worldwide, prostate cancer (PCa) is one of the most common types of cancer in men and the number five leading cause of death [1]. Within five years after diagnosis of primary PCa, 10–20% of the patients have metastatic castration-resistant prostate cancer (mCRPC) [2]. Systemic treatments of mCRPC consists of androgen receptor (AR) or androgen receptor signaling inhibitors (abiraterone or enzalutamide), chemotherapy (docetaxel or cabazitaxel), Radium-223 (for patients with bone metastases only), immunotherapy (sipuleucel-T or ipilimumab) and poly ADP-ribose polymerase (PARP) inhibitors (olaparib and rucaparib) [3]. However, life expectancy of patients with mCRPC is still limited with an overall survival ranging from 3.6 to 34.7 months [4, 5].

More recently, radionuclide therapy (RNT) targeted against prostate-specific membrane antigen (PSMA) has been investigated. PSMA is a type II transmembrane glycoprotein with both an intracellular and extracellular domain [6, 7] and is expressed on benign prostate epithelium and on prostate cancer cells. PSMA is also expressed in other tissues such as the kidneys, small intestine and the salivary glands [6]. However, the expression on prostate cancer cells is approximately a thousand-fold higher than the expression on normal tissues [8]. Therefore, PSMA is a target for both imaging and therapy of prostate cancer. Imaging with Gallium-68 (Ga-68) or Fluorine-18 labeled PSMA ligands is already widely used for primary staging of PCa and for the detection of recurrent disease in patient with a biochemical recurrence [9]. In the recent years, different PSMA ligands were developed for therapy such as PSMA-617 and PSMA Imaging and Therapy (I&T) [10].

In 2022, the Food and Drug Administration approved treatment with Lutetium-177 PSMA-617 (^177^Lu-PSMA-617) in the USA for patients with PSMA-positive mCRPC who had previous treatment with AR inhibitors and taxane-based chemotherapy [11–13]. The results of two randomized trials led to this approval. In the phase II TheraP trial, an improved progression-free survival (PFS) of 5.1 mo was achieved with ^177^Lu-PSMA-617 as compared to cabazitaxel (hazard ratio (HR) 0.63) [12]. The phase III VISION trial compared the radiographic PFS (rPFS) and overall survival (OS) in two treatment arms [13]. In this randomized trial, 831 patients with progressive PSMA-positive mCRPC were treated with ^177^Lu-PSMA-617 RNT and standard of care (SOC) or with SOC alone. An improved rPFS and OS was achieved in the ^177^Lu-PSMA group (8.7 and 15.3 months, respectively) compared to the SOC only group (3.4 and 11.3 months, respectively).

Although ^177^Lu-PSMA-617 improves PFS and OS [12, 13], Actinium-225 (Ac-225) PSMA (^225^Ac-PSMA) might be an even more promising radiopharmaceutical for the treatment of mCRPC [14–19]. Ac-225 is an alpha-emitter with a half-life of 9.9 days and emits four alpha particles upon decay with energies ranging from 5.8 to 8.4 MeV and tissue penetration of 47 to 85 μm [20]. The decay cascade also includes two beta particles and gamma co-emissions, which may be used for imaging [20]. The use of alpha emitting radionuclides has advantages over beta emitting radionuclides, such as Lu-177, since alpha emitting radionuclides have a high linear energy transfer (LET) with a short radiation range [21]. The high LET nature of the alpha particles makes them a potent radiation capable of effectively killing tumor cells by a single or few event(s) through breaks of deoxyribonucleic acid (DNA) double strands and DNA clusters, and the short range allows selective tumor cell killing while reducing the radiation effects in the surrounding healthy tissue [20]. As a result, alpha emitting radionuclides have a more complex DNA damage with less possibility for repair than beta emitting radionuclides [21]. Previously, small retrospective case series showed efficacy and tolerability of ^225^Ac-PSMA-617 in patients with both heavily pretreated and chemotherapy-naïve mCRPC [15–18, 22, 23]. Therefore, alpha emitting radionuclides could provide patients with mCRPC a new treatment option to further improve their survival and quality of life.

As there is still a lack of knowledge on the safety and optimal dose of ^225^Ac-PSMA, prospective phase I trials are needed to determine the optimal dose level. Therefore, a phase I dose-escalation trial was initiated for ^225^Ac-PSMA-I&T in patients with mCRPC to assess the safety and tolerability of ^225^Ac-PSMA-I&T and to recommend an optimal dose for a phase II trial.

## Methods/design

### Study design

**Fig. 1:**
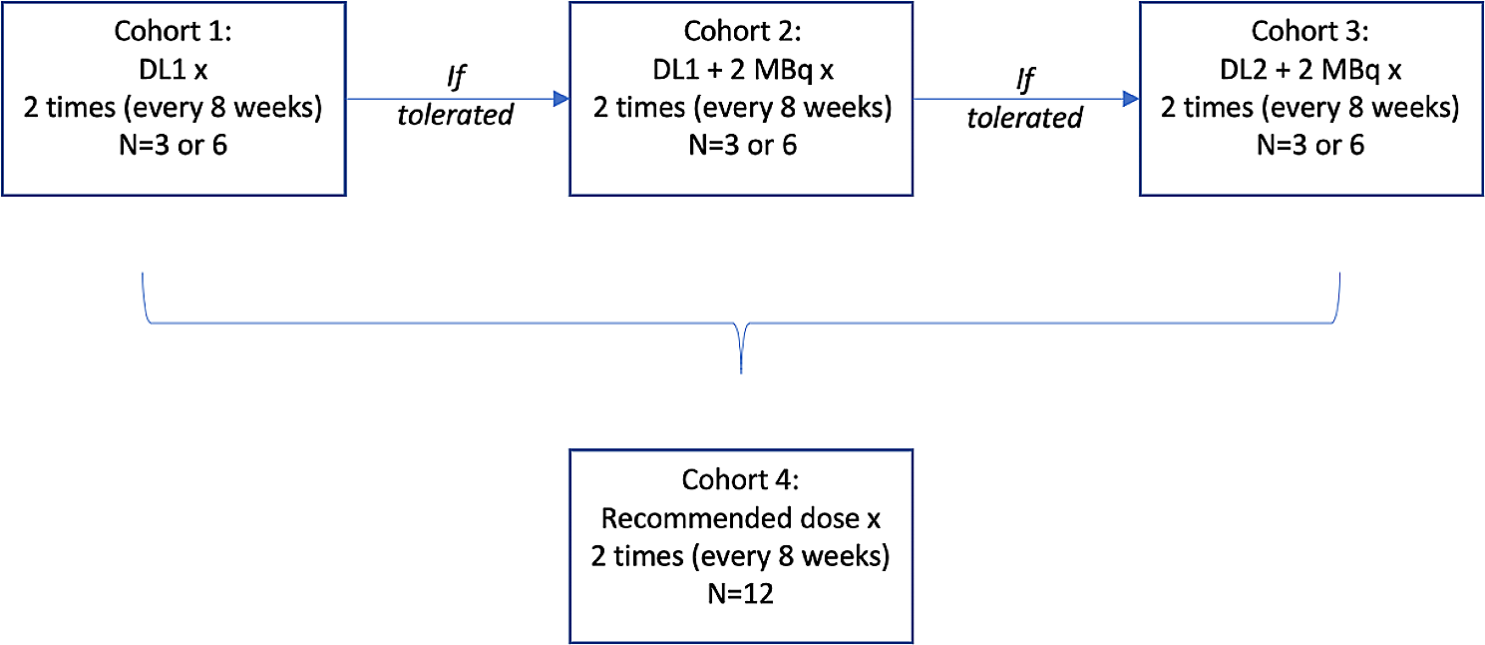
Dose-escalation design of the ^225^Ac-PSMA I&T phase I trial. DL = Dose Level. MBq = Megabecquerel

This is a phase I, single-center, open label, repeated dose-escalation and expansion trial (See Fig. 1) in patients with mCRPC and sufficient PSMA expression (defined as at least one metastatic site with significant increased uptake value as compared to the normal liver uptake) on PET imaging. The dose-escalation will proceed following an accelerated 3 + 3 design which will allow to open the next dose-level cohort in the absence of dose limiting toxicity while the previous cohort is still ongoing. Up to four treatment cohorts will be explored including up to three dose-escalation cohorts (with dose-levels of 8, 10 and 12 MBq, respectively) and a last one (expansion cohort) where patients will be administered with the recommended dose. A total of up to 30 patients will be enrolled in the dose-escalation and expansion cohort. All patients will be evaluated for safety.

Additionally, extensive dosimetry will be performed for the first three patients in cohort 1 after the first administration of ^225^Ac-PSMA-I&T. The feasibility and results of this dosimetry protocol will be evaluated after the first three patients during the trial. If there is significant benefit (determined by a better trade-off between absorbed doses in tumor lesions and the absorbed doses in the critical organs/side effects) for these patients, also the following patients in the dose-escalation will undergo extensive dosimetry. In the case of limited benefit for patients, the dosimetry protocol will be adjusted.

### Study objectives

The primary objective of this phase I trial is to assess the maximum tolerated dose (MTD) and recommended a phase 2 dose based on tolerability and safety of ^225^Ac-PSMA-I&T. The secondary objectives are to calculate the maximum absorbed dose in critical organs (e.g. bone marrow, kidneys, salivary glands) using PET/MRI and whole-body imaging with Single-Photon Emission Computed Tomography / Computed Tomography (SPECT/CT) post-therapy. In addition, short-term changes in quantitative parameters after ^225^Ac-PSMA-I&T will be assessed using PET/MRI with ^68^Ga-PSMA-I&T.

### Patient selection

#### Inclusion criteria

- Histopathological proven metastatic castration resistant prostate cancer. Castration-resistant disease is defined as a serum testosterone level of ≤ 50 nanogram per deciliter or ≤ 1.7 nanomol per liter after bilateral orchiectomy or during maintenance treatment consisting of androgen-ablation therapy with a luteinizing hormone–releasing hormone agonist.
- Evidence of progressive disease, defined as 1 or more Prostate Cancer Work Grouping 3 (PCWG3) criteria: − PSA level ≥ 1 ng/mL that has increased on at least 2 successive occasions at least 1 week apart.
- Progressive disease as defined by RECIST 1.1 according to PCGW3 modifications.
- Progressive disease after at least one treatment line of chemotherapy and/or one line of nonsteroidal antiandrogen.
- No active anti-tumor therapy, except for androgen deprivation therapy in combination with at least one androgen receptor-targeted agent.
- Willing and able to undergo two cycles of ^225^Ac-PSMA-I&T therapy and three PET/MRI scans within 16 weeks according to the protocol.
- Signed and dated written informed consent by the patient (or legal representative) prior to any study-specific procedures.
- Age $$ \ge $$ 18 years.
- Eastern Cooperative Oncology Group (ECOG) performance-status score 0–2.
- Use of highly effective methods of contraception (female partners of male participants) during the trial and 6 months after completion of the study or willing to practice sexual abstinence.

#### Exclusion criteria

- Concurrent severe illness or clinically relevant trauma within 2 weeks before the administration of the investigational product that might preclude study completion or interfere with study results.
- Serum hemoglobin ≤ 6.2 mmol/L, total white blood cell (WBC) count ≤ 3.5·10^9^/L, absolute neutrophil count ≤ 1.5·10^9^/L, platelet count ≤ 100·10^9^/L, serum creatinine concentration ≥ 150 umol/L (≥ 1.7 mg/dL), serum albumin < 30 g/L, bilirubin ≥ 1.5 x upper limit normal (ULN), aspartate aminotransferase (ASAT) ≥ 3 x ULN and alanine aminotransferase (ALAT) ≥ 3 x ULN (or bilirubin ≥ 3 x ULN, ASAT ≥ 5 x ULN and ALAT ≥ 5 x ULN in the case of pre-existing liver metastases at baseline).
- Concurrent bladder outflow obstruction or unmanageable urinary incontinence.
- Known or expected hypersensitivity to Ga-68, Ac-225, PSMA-I&T, or any excipient present in ^225^Ac/^68^Ga-PSMA-I&T.
- Prior administration of a radiopharmaceutical within a period corresponding to eight half-lives of the previously administered radionuclide.
- Prior treatment with any type of radionuclide therapy.
- History of somatic or psychiatric disease/condition that may interfere with the objectives and assessments of the study.
- Central nervous system (CNS) metastases, leptomeningeal disease, or spinal cord compression.
- External beam radiation therapy within 4 weeks of first dose (or local or focal radiotherapy within 2 weeks of first dose).
- Male subjects unwilling to abstain from donating sperm during treatment and for an additional 6 months after the last dose.

### Statistical calculations for trial sample size

The primary objective of the trial is to assess the safety and tolerability of ^225^Ac-PSMA-I&T, to assess dose-limiting toxicities and determine the MTD (if reached) and define the recommended dose for the phase II trial. Since the data for this objective will be descriptive, a formal statistical sample size calculation is not required. The maximum number of patients has been set on 30 patients. This sample size is considered sufficient for answering the research questions of this study.

### Overview of current protocol

#### Definitions of study periods and visits of the phase I study

- **Screening Period**, (Day − 28 to D0) to establish protocol eligibility after obtaining informed consent.
- **Imaging Period**: at the screening visit, at Day 7 after treatment visit 1 and at Day 42 after treatment visit 2, PET-MRI using ^68^Ga-PSMA-I&T will be performed to acquire dosimetry data and for response evaluation. Because of the different energy levels of the radiation, the effect of ^225^Ac-PSMA-I&T on the images of the ^68^Ga-PSMA-I&T PET-MRI is considered minimal and no effect on the signal-to-noise ratio of PET/MRI is expected. When feasible, a whole-body (WB) planar scan will be acquired at Day 7 to image residual ^225^Ac-PSMA I&T. The WB planar image will be acquired before the ^68^Ga-PSMA-I&T PET-MRI or 4–5 h (h) thereafter to prevent any effects of radiation emission by Gallium-68 on the imaging quality of the WB scan.
- **Treatment Visits**: days of administration of ^225^Ac-PSMA-I&T.
- **Safety Visits**: Follow-up period 1: from the end of Imaging period to treatment visit 2, over 8 ± 1 weeks; Follow-up period 2: from the end of treatment visit 2 to the end of study visit (16 ± 1 weeks).
- **Follow-up Visits**, will be performed four times after each cycle and 24 weeks after the first cycle.
- **End of Study Visit**, which will scheduled at 24 weeks after the first administration of ^225^Ac-PSMA-I&T.

Venous blood samples will be collected before and after ^225^Ac-PSMA-I&T administration, at Day 7, Day 14, Day 28 and Day 42 after each cycle. Urine will be collected at the same time points.

#### Study procedures of the dosimetry protocol after the first administration of ^225^Ac-PSMA-I&T

- Blood sampling will be performed at t = 0 (end of infusion), 5, 10, 20, 30 and 60 min, 2, 4 and 8 h and at each imaging time point (24, 48, 72–96 and 168 h).
- Urine samples will be collected over the first 24 h in time intervals: 0–2 h, 2–4 h, 4–8 h and 8–24 h.
- Planar WB scans will be performed at 4, 24, 48, 72–96 and 144–168 h post therapy.
- SPECT/CT scans of the head / thorax (salivary and lacrimal glands in field of view) and the abdomen will be made at 4 and 24 h. Acquisition and reconstruction parameters will be used as published by Benabdallah et al. [24].

### Study endpoints and analyses

#### Primary endpoints

The primary endpoints of this study are: 1) safety and tolerability of ^225^Ac-PSMA-I&T in patients with mCRPC as assessed by, (a) incidence and severity of adverse events (AEs) and serious adverse event (SAEs), (b) absolute values and changes from baseline in laboratory parameters (hematology, blood chemistry and urinalysis), including assessment of shifts from baseline to abnormal values on treatment and (c) Absolute values and changes from baseline in vital signs & electrocardiography (ECG) parameters, and 2) a recommended dose for further phase II study.

#### Analysis of the primary endpoints

The evaluation of the safety of ^225^Ac-PSMA I&T will be based on the analysis of the incidence, severity, type and consequences of each AE/SAE. Also, all clinically significant value changes of the vital signs, laboratory values and ECG parameters will be reported. AEs and SAEs will be presented on a per patient basis and will be subdivided per organ classes. Descriptive tabulation will be presented for all AEs and SAEs on the incidence, type, severity and possible relation to the investigational product.

Categorical data will be presented as absolute and relative frequencies. Continuous data will be presented as means (and standard deviations) or medians (and interquartile ranges). The observed baseline values and changes over time of vital signs, ECG parameters and laboratory values will be presented graphically using boxplots.

#### Secondary endpoints

The secondary endpoints include the following:

- the volume calculations of critical organs and tumor using ^68^Ga-PSMA-I&T PET-MRI.
- the expected radiotracer uptake in critical organs and tumor calculated as a percentage of the injected dose per gram of tissue (%ID/g).
- the expected absorbed doses by ^225^Ac-PSMA-I&T in critical organs and tumor.
- the changes in maximum Standardized Uptake Value (SUVmax) of the target lesions on ^68^Ga-PSMA-I&T PET-MRI.
- the morphological changes on MRI by measuring the changes in MRI signals.
- the objective response rate (ORR) as measured by Response Evaluation Criteria in Solid Tumors (RECIST) criteria v.1.1.
- the percent changes from baseline in tumor size where tumor size is defined as the sum of all target lesions as measured by RECIST criteria v.1.1.
- the PSA response rate assessed from treatment visit 1 defined as a decrease in PSA of ≥ 50% from baseline.
- the percent change from baseline in PSA as a continuous endpoint by visit and maximum reduction during the study.
- the OS defined as the time from the date of first dose of ^225^Ac-PSMA-I&T treatment to the date of death due to any cause.
- the percent change from baseline values of pain questionnaire (Brief Pain Inventory short version, Dutch language) at every treatment visit.

### Analysis of secondary endpoints

Preliminary efficacy analyses will be performed for all patients in the study. Objective response rate using the RECIST 1.1 will be measured for patients with visceral disease at baseline and will be presented with its 95% confidence intervals. The numbers of patients with either complete response, partial response, stable disease or progressive disease according to RECIST 1.1 will be summarized descriptively.

Changes in tumor size at the end of the study from treatment visit 1 will be summarized descriptively for patients with measurable disease by RECIST at baseline for patients. This data will also be displayed graphically in waterfall plots which present vertical bars representing each patient’s % change from baseline in tumor size ordered from largest increase to largest decrease, including reference lines at + 20% and − 30% (indicators of target lesion progression and response per RECIST guidelines).

PSA response rate with 95% confidence intervals will be presented by dose group. Changes in PSA will also be summarized descriptively.

An exploratory analysis will be performed to explore any preliminary efficacy of ^225^Ac-PSMA I&T as detected from ^68^Ga-PSMA I&T imaging. Summaries of number and location of lesions as detected by ^68^Ga-PSMA I&T imaging and SUV values will be produced at screening and at the end of the study to assess change in the number of lesions with uptake of^68^Ga-PSMA I&T and the change in SUV values. Any subject not known to have died at the time of the analysis will be censored based on the last recorded date on which the subject was known to be alive.

### Monitoring

Monitoring is performed according to good clinical practice guidelines by the Clinical Trial Center of the Erasmus Medical Center, Rotterdam, the Netherlands.

### Trial status

The trial is open for inclusion since March 2022 and is estimated for completion in December 2024. The approved protocol is version 5, 5 December 2022. This trial is retrospectively registered at ClinicalTrials.gov on 13 June 2023 (NCT05902247).

## Discussion

Although ^225^Ac-PSMA therapy showed promising results, current clinical data are only based on retrospective studies in relatively small groups of patients with mCRPC [19]. For the assessment of the safety and tolerability of ^225^Ac-PSMA therapy, more prospective data are needed. In this investigator-initiated phase I trial, we aim to assess the safety and tolerability of ^225^Ac-PSMA I&T in patients with mCRPC and to recommend the optimal dose for a phase II trial.

For imaging in the current trial, a hybrid Positron Emission Tomography / Magnetic Resonance Imaging (PET/MRI) scanner is used. As metastases in patients with mCRPC are most commonly detected in the bones, lymph nodes and liver, the PET/MRI is considered more sensitive to detect metastases due to better visualization of soft tissues and better characterization of the liver and bone lesions [25–27] (See Fig. 1).

**Fig. 2:**
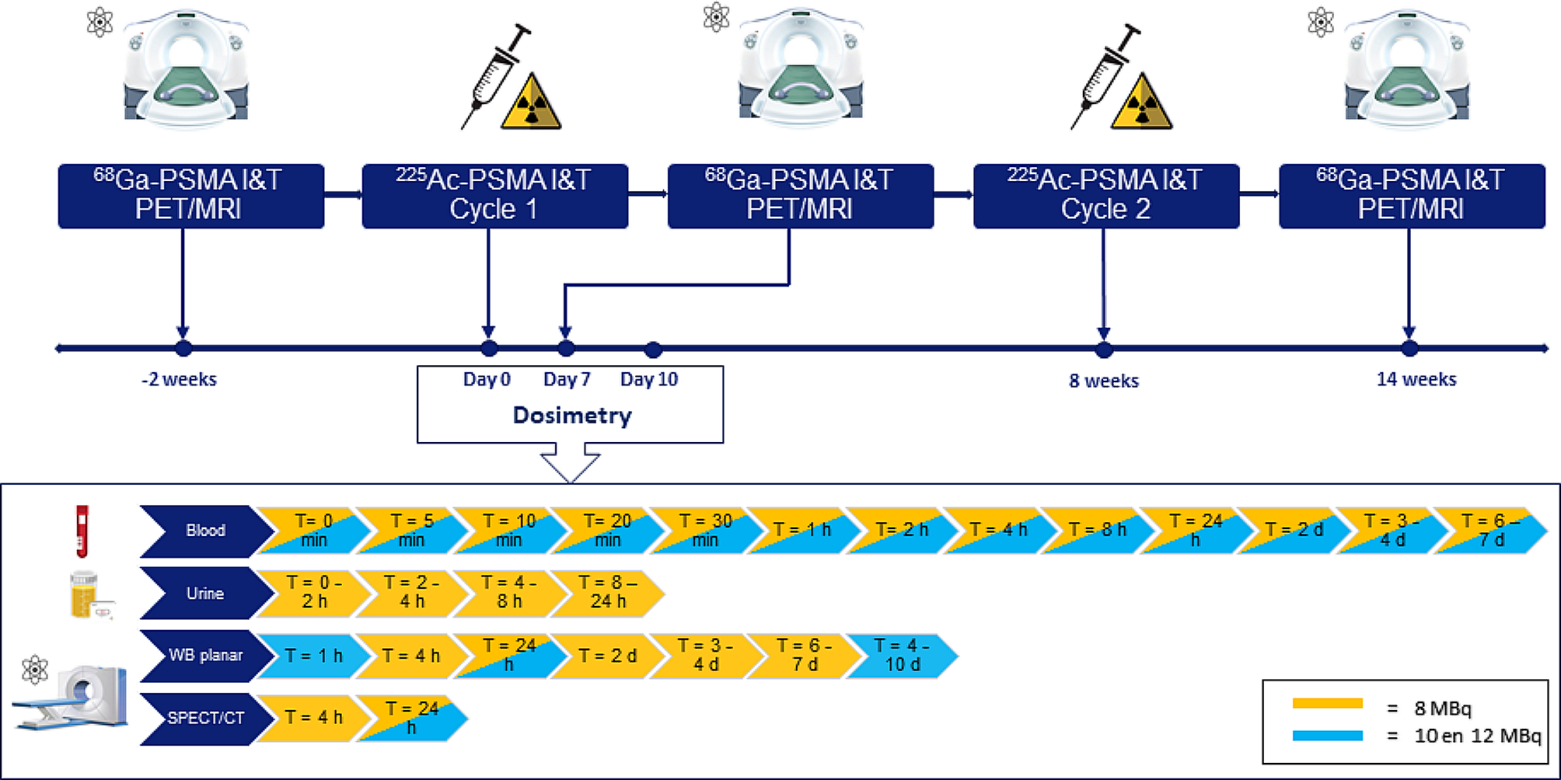
Study protocol and dosimetry protocol (dosimetry will be performed for the first three patients in cohort 1 after the first administration of ^225^Ac-PSMA I&T). ^225^Ac = Actinium-225. d = Day. ^68^Ga = Gallium-68. h = Hour. I&T = Imaging & Therapy. Min = minute. MBq = Megabecquerel. PET/MRI = Positron Emission Tomography / Magnetic Resonance Imaging. PSMA = Prostate-specific Membrane Antigen. SPECT/CT = Single Proton Emission Computed Tomography / Computed Tomography. Wb = Whole-body. Adapted from 123RF by captainvector.

PSMA is not as prostate specific as the name would suggest since many other tissues such as the kidneys, small intestine and the salivary glands have PSMA expression [6]. To predict toxicity on these healthy tissues, it is essential to calculate the absorbed dose prospectively and compare these data with previous published data. Since Ac-225 is an alpha emitting radionuclide, dosimetry assessments direct after therapy are challenging. Nevertheless, it is still necessary to calculate the absorbed doses to determine to what extent the tumors and healthy critical organs (e.g. bone marrow, kidneys, salivary glands) are affected by the radiation. Therefore, we designed an extensive dosimetry protocol, but this protocol was adapted after evaluation of the first three patients due to the limited benefit and high burden for patients. In the reduced dosimetry protocol, blood sampling remained unchanged and urine sampling was discontinued. WB planar imaging was reduced from five time-points to three time-points at 1 h, 24 h and 4–10 days. SPECT/CT imaging of the head and abdomen was reduced from two time-points to one time-point at 24 h (See Fig. 2).

The current study is the first phase I trial for ^225^Ac-PSMA-I&T therapy under full Good Manufacturing Practice compliance. Since the same PSMA ligand is used for both imaging and therapy, it is expected that ^68^Ga-PSMA-I&T and ^225^Ac-PSMA-I&T have the same uptake pattern in healthy tissue and tumors. As a result, the biodistribution of ^225^Ac-PSMA-I&T therapy can be predicted very precisely. In most studies on ^225^Ac-PSMA, patients with mCRPC were treated with ^225^Ac-PSMA-617 [15–17, 22, 23], while only one clinical study investigated ^225^Ac-PSMA I&T [18]. Preclinical and clinical data have showed that both PSMA ligands, when labeled with ^177^Lu or ^225^Ac, have a comparable in vivo behavior, toxicity profile and efficacy in patients with mCRPC [28–31]. For therapy with PSMA-617, however, a different diagnostic ligand (PSMA-11) is usually used for imaging since PSMA-617 is not suitable for diagnostic purposes. In clinical practice, this is not a problem because of the excellent representation of PSMA expression with both PSMA-I&T and PSMA-11 [32].

In conclusion, to assess the safety and tolerability of ^225^Ac-PSMA-I&T in patients with mCRPC and to recommend the optimal dose for a phase II trial, the design of the current phase I trial includes imaging using PET/MRI, an extensive dosimetry protocol, and use of the same PSMA-I&T ligand for imaging and therapy.

## Data Availability

No datasets were generated or analysed during the current study.

## Abbreviations

%ID/g: Percentage of the Injected Dose per Gram of tissue
^225^Ac: Actinium-225
AE: Adverse Event
ALAT: Alanine Aminotransferase
AR: Androgeen Receptor
ASAT: Aspartate Aminotransferase
CNS: Central Nervous System
CT: Computed Tomography
DNA: Deoxyribonucleic Acid
ECG: Electrocardiography
ECOG: Eastern Cooperative Oncology Group
^68^Ga: Gallium-68
h: Hour
HR: Hazard ratio
I&T: Imaging & Therapy
LET: Linear Energy Transfer
^177^Lu: Lutetium-177
mCRPC: metastatic Castration-Resistant Prostate Cancer
MeV: Megaelectronvolt
µm: micrometer
mmol: Millimol
mo: Month
MRI: Magnetic Resonance Imaging
MTD: Maximum Tolerated Dose
OS: Overall Survival
PARP: Poly ADP-ribose Polymerase
PCa: Prostate Cancer
PCWG3: Prostate Cancer Work Grouping 3
PET: Positron Emission Tomography
PFS: Progression-free Survival
PSMA: Prostate Specific Membrane Antigen
RECIST: Response Evaluation Criteria in Solid Tumors
RNT: Radionuclide Therapy
rPFS: radiographic Progression-free Survival
SAE: Serious Adverse Event
SOC: Standard of Care
SUVmax: maximum Standardized Uptake Value
ULN: Upper Limit Normal
WB: Whole-body
WBC: White Blood Cell
